# A rabbit is not a mouse: a framework for the initial welfare assessment and long-term monitoring of genetically altered laboratory rabbits

**DOI:** 10.3389/fvets.2026.1831790

**Published:** 2026-07-02

**Authors:** Sara Fuochi, Alessandro Cozzi, Alessandra Bergadano

**Affiliations:** 1 Department of Ethics, Legislation & Animal Welfare, Research Institute in Semiochemistry and Applied Ethology (IRSEA), Apt, France; 2 Experimental Animal Center (EAC), University of Bern, Bern, Switzerland

**Keywords:** animal models, framework, genetically altered, phenotype, rabbit, welfare

## Abstract

Genetically altered (GA) rabbits are used in biomedical research due to their unique physiological and translational relevance. Despite their importance in multiple biomedical research specialties, species-specific guidance for their welfare assessment is limited. This article proposes a comprehensive framework for the initial and long-term welfare evaluation of GA rabbits, covering all life stages from birth to aging. Key indicators and biomarkers include neonatal viability, maternal care, post-weaning growth, sexual maturation, reproductive performance, and late-onset phenotypes, alongside clinical, behavioral, and physiological markers. Emphasis is placed on recording evidence to contextualize observed deviations from wild-type baselines. Accurate monitoring of phases-specific biological biomarkers enables further detection of relevant phenotypes, beyond initial welfare assessment. This framework offers practical guidance for researchers, veterinarians, animal welfare officers and competent authorities, promoting not only non-maleficence but also positive welfare approaches. Implementing a species- and model-specific frame facilitates responsible management of GA rabbits, ensuring ethical integrity, scientific rigor, and the development of positive animal welfare strategies throughout their lifespan.

## 1 Introduction

In recent years, the use of genetically altered animals (GAA) has become increasingly important in biomedical research. In 2017, genetically altered animals accounted for nearly one-third (2.59 million) of all animals used in scientific research and testing within the EU (1). Mice (38%) and zebrafish (64%) were the most reported GAA species, although significant numbers of Xenopus, rabbits, and rats were also reported, along with smaller numbers of other species such as guinea pigs, dogs, pigs, sheep, domestic fowl, and various fish species (1).

The latest EU statistical report (2), summarizing data from the EU and Norway for 2023, reports a total of 7,974,226 animals used. The use of rabbits increased from 332,097 animals in 2018 to 372,239 in 2022 (+4.4% from 2021 to 2022 and +12.1% from 2018 to 2022), although a slight reduction was recorded in 2023, with 349,055 rabbits reported (still +5.1% compared with 2018) (2, 3). If we focus on GAA, mice still represent the largest group of animals being genetically altered, but proportionally they are not. In terms of trends since 2018, the proportion of GA zebrafish has increased by 15.2 percentage points, whereas the proportion of GA rabbits has decreased by 3.5 percentage points (2). Furthermore, in the EU, the use of animals for the creation of new genetically altered lines has shown a reduction in the number of rabbits used, decreasing from 324 in 2018 to 216 in 2022 and 166 in 2023, corresponding to a decrease of 23.1% from 2022 to 2023 and 48.8% from 2018 to 2023 (2, 3). Despite the overall reduction in animal use in the EU and UK (2–4), GA rabbits still account for 4% of all rabbits used in research in the EU in 2023 and remain a non-negligible component in several research areas, contributing to scientific advances and publications both within and outside the EU. The EU commission working document on GAA, published in 2022 (1), does not include specific indications or templates for the welfare assessment of GA rabbits despite being a relevant model in biomedical research. Instead, it provides explicit templates for other species, such as GA rodents, pigs, fish, and poultry. Because rabbits exhibit unique biological and physiological traits that may require specific welfare consideration, it is important that attention is given to the techniques of production and maintenance of such animals, and to the species-specific consequences of genetic alteration in order to apply the three Rs principles not only in the creation, but also in breeding, use and care practices.

Historically, GA rabbits have been less commonly used in research compared to mice or zebrafish, primarily due to intrinsic technical and biological complexities such as longer gestation periods, more challenging reproductive management and more difficult genetic material manipulation (5–7). However, recent advancements in genetic engineering techniques have increased the feasibility of creating rabbits as models for biomedical research, highlighting their unique translational potential (8, 9). GA rabbits are consistently used as models for a wide range of biomedical applications, including cardiovascular research, metabolism, aging, immunology and drug development, among others. These models have proven particularly valuable in studies of cardiac diseases such as long QT syndrome (10–12), or ion channel dysfunctions *in vivo* (13). GA rabbits are also used to investigate atherosclerosis (14), obesity (15), bone physiology (16) and the development of targeted therapies for infectious diseases (17). Additionally, their utility in gene editing and transgenic model research continues to expand, contributing to advancements in understanding complex biological processes and potential therapeutic interventions in areas such as cardiovascular diseases, coagulopathies and immunotherapy (18–21). Extensive overviews of the vast application of GA rabbits in modern biomedical research are provided by Christensen et al. (22) and Han et al. (7) covering disease types, targeted genes and editing technologies applied.

Considering the specific research areas in which GA rabbits are used, and the increasing attention that national and supranational competent authorities devote to the generation, maintenance, and use of GA models in research, we propose a species-specific framework for the welfare assessment of GA rabbits to bridge a gap in the existing EU framework (1). The main goal of our work is to provide hands-on support for veterinarians, animal caretakers, researchers involved in the generation, breeding and use of such models as well as to animal welfare officers, ethics committees and competent authorities called to take decisions on severity classification and projects approval.

The proposed framework was developed through a targeted narrative review of scientific and regulatory literature on rabbit welfare assessment and genetically altered animal monitoring. This approach consisted in structured searches across PubMed, Web of Science, Scopus and ResearchGate followed by screening and interpretation of selected peer-reviewed literature, regulatory and statistical reporting documents from the European Union, the United Kingdom and Switzerland. Search terms included, but were not limited to, keywords like rabbit, welfare, assessment, behavior, clinical, scoring, pain, biomarkers, phenotype, harmful phenotype, ageing, breeding, production, male, female, genetically modified, transgenic, genetically engineered, and laboratory animals. Terms were used in multiple combinations across databases to identify relevant scientific and regulatory literature supporting framework development across multiple domains and life stages. The literature search was used to identify relevant scientific and regulatory evidence to support framework development across multiple domains.

Evidence prioritization favored peer-reviewed literature and regulatory guidance directly applicable to laboratory rabbits and genetically altered animals. Web pages and technical datasheets from professional breeders were also consulted to support the characterization of and comparison with wild-type background strains. Additional literature from production and pet rabbit medicine was included where laboratory rabbit-specific evidence was limited or missing and integrated with expert input from laboratory animal welfare practice This approach was adopted intentionally to overcome the compartmentalization of knowledge across laboratory animal science, pathology, production and companion animals’ clinical domains. Assessment of the impact of genetic interventions in a defined species should be grounded in an understanding of the species as a whole. We adopted a holistic approach to species-specific welfare assessment, not limited by context of use and therefore, integration of multidisciplinary knowledge was considered essential. Indicators and monitoring approaches were then selected based on biological plausibility, consistency across multiple evidence sources, and relevance to practical welfare assessment in laboratory settings.

## 2 Welfare assessment frame for GA rabbits

Welfare assessment is crucial to ensure that genetically altered animals are monitored, understood and treated humanely, with the objective of minimizing pain, distress, and long-term suffering. The weighing of potential phenotypes against normal physiology acknowledges the GA animals’ dignity. This assessment process evaluates not only the health of the animals but also their capacity to engage in normal biological functions and reproductive behaviors, to confirm or exclude the presence of any harmful phenotypes – which would frame the management of the GA strain under the ethical and legal perspective. All the key general principles highlighted in the EU framework on GA mammals, as reported under section A of the EU commission working document (1), can already serve as a guiding reference for a more specific welfare assessment pipeline for GA rabbits. According to the framework, the first step of this multiple-species template to track and report the welfare assessment results is to clearly define the species and the specific line being assessed. This includes specifying both the internal name used within the housing facility, along with the strain number, if applicable, and the international name, which should conform to internationally recognized nomenclature standards. This point, too often neglected or underestimated also during peer review, highlights the crucial relevance of registering official nomenclature for the specific genetic construct the animals carry, as well as the official nomenclature of the strain itself. Official nomenclature allows everyone to understand the terms of genetic alteration, the history of the strain carrying it, and most importantly in the light of the welfare assessment of a GA animal, its genetic background. Reporting official nomenclature, of both genetic alteration and GA animal strain, should be a fundamental part of Materials and Methods section of any research article presenting results deriving from a GA model and should be scrutinized with competence in the peer review process. For the rabbit, the first comprehensive database was published in 2018 (23).

Since mutant animals are compared with wild-type controls, knowledge of the original strain in which the genetic alteration was established provides essential baseline parameters for biomarker comparison. Again, background strain information should be considered, as this offers context about the genetic foundation of the animals in question. The type of genetic alteration should be clearly described as well, including the technique used, the specific genetic target, and how the alteration fits within the broader context of genetic research. Once more, identifying the wild-type genetic background is crucial to understanding how genetic modification may differ from naturally occurring variants. At the time of assessment, it is essential to document detailed information about the animals, including their age, numbers, and sex distribution. The breeding strategy of the assessed animals is another critical element to document. It is important to indicate the preferred breeding schemes based on zygosities and their crosses for colony maintenance. Understanding these breeding practices helps in evaluating reproductive performance, such as pregnancy/mating rate (gender specific), embryonal mortality, the average litter size (LS) and pre-weaning mortality, especially when compared to wild-type controls. This information helps assess how the animals’ age or physiological status impacts their welfare over time. In parallel, housing conditions play a significant role in welfare assessments. Factors such as the type of housing, lighting regime, temperature, humidity, and the environmental enrichment provided in the cages should be recorded. Particularly for the rabbit, relevant information concerning the type of diet should be provided, as food regime can significantly modulate behavior, microbiome, performance, growth, health and survival in this species (24–27). Lastly, any additional relevant information that may impact on the welfare assessment must be included: factors such as changes in environmental conditions, construction work, staff turnover, or health status at the time of the assessment. Such details ensure a comprehensive evaluation of all factors influencing animal welfare. Where available, other relevant publications and websites can be cited to support the assessment, providing broader context and references. This should include, for example, reference to the repository where the genetic alteration and GA strain have been registered. All the findings and records are to be gathered in so called strain passports, or strain data sheets, documents carrying all the relevant information contributing to a broad understanding, management, maintenance, use, dissemination and coherent publication of the GA line.

Going beyond such valuable but general considerations, that are – to some extent - more aligned with post initial assessment reporting rather than initial welfare assessment hands-on best practices, a tailored pipeline for genetically altered rabbits can prove particularly relevant to correctly interpret any deviation from their specific behavioral and biological peculiarities, including maternal behavior, litters viability, normal growth, life expectancy and common clinical conditions.

It is important to remark that when referring to the expression *welfare assessment of GA animals*, the term *assessment* refers to a comparison with their non-GA counterparts. In some respects, thus, the baseline state of wild type subjects is considered the benchmark standard of welfare to use as a comparison, and *welfare* of a GA animal in this specific context is the absence of deviations from WT counterparts. To some extent, and debatably, pre-existing conditions in the background line do not necessarily play a role in the welfare assessment, if their incidence in GA animals is consistent with that in WT animals and efforts are made to reduce the incidence of the problematic trait by selective breeding (1). In this sense, as of today, the definition of welfare of GAA may not always match states or situations where animals are completely healthy - meaning free from occasional harmful traits - within the time of interest (e.g., initial welfare assessment or long-term monitoring). Conversely, the concept of welfare for GA animals is very peculiar and its assessment is challenging, consistently with the difficulty of correctly determining a state of welfare when no univocal definition of such state is acknowledged (28).

This work provides structure beyond theory, proposing welfare indicators representing the operational domain in which welfare assessment is conducted in accordance with regulatory requirements (29). They comprise high-level clusters of animal-based, resource-based, and management-based parameters used to evaluate the overall welfare state of genetically altered rabbits, including for example clinical, behavioral, husbandry-, and colony management-related aspects. Welfare indicators are then further developed, discussed, contextualized, and eventually suggested as potential biomarkers (30, 31) when translated into measurable physiological (e.g., reproductive performance, growth and development), behavioral (e.g., emergence of impaired maternal behavior, aggression), or clinical parameters (e.g., frequency and onset of overt clinical conditions, frailty phenotype) which deviation from normality (that is versus wild-type) is associated with underlying biological processes and capable of providing more specific information on the animal’s biological state and on potential welfare impairment resulting from genetic intervention.

### 2.1 From birth to weaning

The transition from birth to weaning is a critical period in the life of a rabbit, during which various biological, environmental, and genetic factors can significantly impact the health and survival of offspring. Rabbit maternal behavior can be characterized as absentee mothering (32). The doe typically leaves the nest immediately after giving birth to the last kit and does not clean or retrieve any kits that may become separated from the litter. Nursing is minimal, with the doe nursing her kits once a day for approximately 3 to 5 min (33, 34). For GA rabbits, this phase requires careful attention, with a clear focus on species-specific indicators of pups’ viability and maternal care. A crucial analysis in this life stage is the assessment of any neonatal disorders, including signs of starvation, mismothering and cannibalism as well as housing specific risk factors exposure (35). During early life, survival of kits requires an adequate environment, including a well-built nest in a separate section of the mother’s living environment. For newborns in the nesting phase, low individual birth weight is a hazard for mortality because of hypothermia, injuries, starvation and overall weakness (35). A minimal birth weight threshold, under which the survival chance of kits is low, can range from 43 (36) to 50 grams (37), while weights above 75 grams suggest a very high probability (90%) to survive until weaning (38) with reported relevant impact due to genetic background (35), Inadequate nesting behavior of the doe has also been linked with cannibalism risk, particularly when does did not introduce hair or straw into the nest box (39). Cannibalism can also occur when the mother, after swallowing the placenta, also partially eats and fatally injures the kits or due to environmental stressors, although in the laboratory rabbit cannibalistic events appear to be rare (35, 40).

Litter size (LS) also has an impact on newborn rabbits’ survival. Larger LS at birth comes with increased heterogeneity of kits’ weight and decreased average kit weight (35, 41), even if other factors can play a role including fetus position in the uterine horn, the body weight and parity of the does, with nulliparous females kindling smaller kits than multiparous does (42). Efforts to standardize litter are common practices in production rabbits, by improved selection and cross-fostering around post-natal day (PND) 1–3 (35, 43). Due to the extremely short permanence of the doe in the nest to provide milk, without consolidation of kits weight or litter size, smaller and weaker kits may die because stronger and heavier kits suckle a higher share of milk during the short daily suckling events (35, 44, 45). Litter consolidation could prove useful for GA rabbits too, provided the existence of enough milking does and litters synchronized at the same time. In biomedical research facilities, this can be difficult to achieve due to colony sizing and breeding schedules that must be ethically oriented to the 3RS principles to avoid surplus animals. Having multiple females providing care to multiple litters of similar age in the same cage or enclosure, a common and effective strategy in mouse breeding (46) less applicable in rabbits, despite non-exclusive nursing is described (33). In fact, neonatal mortality is generally higher when does are housed in groups compared to when they are kept in individual cages. This is primarily because does that injure or cannibalize their own kits cannot transfer this behavior to the kits of other does when exclusive nursing is practiced. Additionally, stressed or inexperienced does, as well as rabbits housed together may also severely injure or cannibalize the kits (34, 35). The authors’ practical experience (47) indicates that co-housing of does is feasible in biomedical facility settings without apparent adverse effects on litter welfare, growth, or survival outcomes when smart housing systems are used that allow each mother individual access to the nest. In this context, a commercially available microchip-controlled access system (Sure Petcare) was used (48); this reference is included solely to describe the equipment employed and does not represent further scientific evidence supporting the observation. Web applications can also be implemented to remotely control and register access to the nest, as well as food and water consumption and other relevant parameters (47).

It becomes evident then that failure to accurately detect any species-specific deviations in this phase can compromise welfare assessment, timely detection of a potential harmful phenotype and the implementation of appropriate corrective measures. In the light of the above discussion, two aspects are crucial in the welfare assessment of GA rabbits: confirming maternal care appropriateness and kits viability, particularly by confirming the kits’ capability to suck milk, thus survive and progressively and steadily gain weight. Milk intake cannot be assessed via the presence of milk spot, as with neonatal rodents. Regular weight check of litter should be considered a relevant part of pre-weaning welfare assessment, carried out at a frequency that avoids overburdening both the animals and the personnel involved. The most effective timing is before and after nursing events, especially when poor mothering or retarded growth cannot be excluded (35, 49, 50). Ideally, a weighing plate or module could also be considered to provide continuous litter weight data without disturbing mother-litter privacy, though it would require caging system customization. Easily accessible nesting areas can allow regular check, without disrupting female caring behavior and nest environment and dynamics, and avoiding arousing nest defense triggers (32, 33). Daily checks, as required by guidelines and regulatory frameworks to ensure that all sick or injured animals are identified and appropriate action is taken (51–53), can be more feasible and less disruptive of the nest balance in rabbits than with rodents, once again due to the specific maternal behavior of the doe, attending the nest for a very short time. This also allows for accurate monitoring of any lost kit from birth to weaning. Careful and timely recording of rearing loss is crucial in the assessment of GA animals, and rabbits too, to identify any early-life lethal factors. When dead kits are found in the nest, at the very least a genetic screening to assess the genotype should be performed, to investigate any correlations between specific zygosities and death. Full necropsy, including pathology, should also be performed to further investigate the presence of unexpected abnormalities that could not be overtly observed. Highest mortality, or the first wave of losses, tends to occur in the first 7–10 days of life (49, 54). Later mortality, occurring before or around weaning and in the absence of obvious weight, growth, or gastro-intestinal (GI) issues, may suggest conditions more related to the genetic alteration than to mortality aligned with species-specific risks. Relevant clinical conditions for pre-weaning rabbits are adapted from Rashwan et al. (55), Nowland et al. (56) and Ozawa and Gleeson (34) based on pondered relevance consistent with SPF and SOPF hygiene levels of modern laboratory animal facilities. For conventional or lower hygiene levels, further clinical conditions related to GI and respiratory infections or infestations should not be ruled out *a priori*. Finally, the sex ratio and zygosity of the kits should be carefully documented to ensure that the breeding scheme is yielding the expected genetic outcomes. A skewed sex ratio or abnormal zygosity distribution may suggest genetic anomalies or unintended genetic consequences resulting from the genetic modification. In this phase, it is important to confirm that genotypic ratios (e.g., heterozygote vs. homozygote) align with what is expected from the breeding strategy used. Consistent deviations from expected ratios of genotypes and sexes should suggest that lethal factors might be associated with genetic intervention. Other biomarkers, oriented toward gross appearance like self-feeding, eyes and ears opened, time to transitioning from sleek fur coat to guard hairs fully grown (34) can be considered too, in early developmental phases.

In Table 1, using the same schematic structure as proposed in the Framework for the GAA under Directive 2010/63/EU - Section B template for Rodents (1), we propose the essential components and parameters for assessing the welfare adapted to rabbits from birth to weaning.

**Table 1:** Specific considerations for welfare assessment of neonatal rabbits up until weaning.

Criteria	What to look for
Clinical conditions	Congenital conditions (e.g., hydrocephalus, malocclusion*, cardiac defects, microphthalmia, anophthalmia, buphthalmia/congenital glaucoma). Delayed development (e.g., eye opening beyond PND10). Impaired mobility, splay leg. Gastrointestinal disorders*, signs of nutritional imbalance*. *More detectable around weaning age.
Behavioral signs	Reactivity/jumping behavior, search and orientation towards doe/nipples, nipple attachment and suckling, lethargy/unresponsiveness, huddling.
Weight and weight gain	Low weight at birth, failure to gain weight (runt), growth retards.
Maternal behavior	Nesting behavior (e.g., fur pulling, nest building); Evidence of poor mothering (cannibalism, reluctance to enter the nest and/or nurse). Milk output.
Litter quality	Litter size, litter homogeneity, kits viability and day-to-day mortality, overall pre-weaning losses. Lethal factors (missing or under-represented sex or genotype).

### 2.2 Post-weaning

Post-weaning is a complex phase in the life of a rabbit, escorting kits out of their infancy, with timeline differences based on rabbits’ strains and purpose. With reference to laboratory rabbit strains, weaning is normally performed after the 4th week of life, more commonly around week 5, but no later than week 8 (57). Here we will consider different life stages of the laboratory rabbit, mostly using the New Zealand White (NZW) strain as baseline reference, being the most used laboratory strain, from end of infancy to puberty, up to sexual maturity and early and late adulthood, highlighting key biomarkers that could be of relevance for the welfare assessment of GA rabbits. Criteria, welfare indicators, developmental, physiological and behavioral biomarkers applicable to juvenile, adult and breeding rabbits are summarized in Tables 2, 3.

**Table 2:** Specific considerations for welfare assessment of juvenile rabbits up to sexual maturity.

High-level category	Criteria	What to look for
Growth and physical development	Weekly growth curve	Consistent weight gain comparable with WT littermates, colony standards, and published strain references, i.e., 3rd ≤ percentile ≤ 97th on standardized growth chart; any deviation from expected developmental trajectory;
	Body weight at weaning	Minimum thresholds reached before weaning (e.g., ~500 g at week 4; ~800 g females and ~900 g males at week 5 for NZW- standards)
	Post-weaning recovery	Improvement in size and condition after delayed weaning in underweight rabbits; adequate adaptation to solid diet
	Daily weight gain	Appropriate breed-specific growth rates (e.g., NZW ~ 15–20 g/day); slowed or excessive gain compared with expected standards
	Cumulative growth progression	Persistent divergence from expected growth curves across juvenile development
	Body length and skeletal growth	Delayed or disproportionate body development; reduced skeletal growth compared with WT or reference standards, morphological abnormalities.
Developmental and reproductive maturation	Sexual maturation	Delayed onset of puberty or reproductive maturity relative to breed expectations and body condition
	Sex-related growth differences	Abnormal divergence between male and female growth or maturation patterns
Environmental and husbandry factors	Housing-density effects	Reduced growth or altered development associated with housing and social conditions
	Diet-related confounding factors	Clinical changes associated with feed changes between breeding and experimental facilities rather than genotype
Clinical monitoring	Gastrointestinal adaptation	Reduced feed intake, diarrhea, bloating, altered feces, showing poor adaptation during dietary transition after weaning
	Emerging genotype-associated phenotype	Early model specific clinical signs expected from the genetic alteration becoming overt during juvenile-to-adult transition
	General welfare status	Poor body condition, lethargy, failure to thrive, reduced activity, or increased morbidity/mortality compared with controls
Behavioral assessment	Behavioral development	Expected mild territoriality/puberty-associated aggression versus abnormal frequency or severity of aggression
	Aggressive or abnormal social behavior	Escalating aggression, injury, persistent fighting, social instability, or behaviors inconsistent with normal puberty
	Founder-related behavioral traits	Recurrent undesirable behavioral phenotypes potentially linked to founder selection rather than intended genotype

**Table 3:** Specific considerations for welfare assessment of adults and breeders.

High-level category	Criteria	What to look for
Reproductive efficacy, fertility, breeder-related variables	Fertility, pregnancy outcome, and fetal viability assessment	Repeated unsuccessful matings, absence of pregnancy, ultrasound evidence of failed pregnancy or embryonic mortality, impaired fetal development, or failure to produce live kits increased stillbirths, consistent infertility associated with a specific sex or GA genotype when crossed with fertile WT controls;
	Reproductive success indicators	Reduced pregnancy, kit index or kindling rates/doe compared with WT or colony standards;
	Reproductive lifespan and breeder performance	Premature decline in fertility, shortened breeding lifespan, or reproductive impairment associated with breeder age, parity, genotype.
	Breeding strategy and mating method	Differences in reproductive outcomes associated with natural mating versus artificial insemination or with breeder selection practices
	Offspring outcomes	Smaller litter sizes, reduced total or live-born kits, or abnormal variation compared with WT or historical colony data
	Parental care and kit survival	Poor maternal behavior, reduced nursing, aggression toward kits, neglect, or increased neonatal mortality
Colony-level performance	Longitudinal colony reproductive performance	Progressive decline, instability, or persistent deviation from WT and historical colony reproductive benchmarks
	Corrective breeding management needs	Trends suggesting founder effects, colony drift, reduced sustainability, or the need for selective breeding interventions
Environmental and husbandry factors	Environmental and husbandry influences	Reproductive deviations potentially linked to housing, diet, feed intake, temperature, light cycles, hygiene status, microbiome, or transfer between institutions
Clinical monitoring	Female reproductive tract health	Presence of ovarian, oviductal, or uterine cysts, ectopic gestation, uterine ischemia, or other gynecological abnormalities
	Male reproductive tract health	Tubular atrophy, Leydig cell atrophy, squamous metaplasia, or other spontaneous reproductive lesions
	Any non-specific clinical conditions	To be assessed against expected and unexpected GA-related phenotypes, as well as possible background-related effects.

#### 2.2.1 Juvenile rabbits up to sexual maturity

In this phase of the rabbit’s life, which is characterized by the challenges of post-weaning adaptation, transitioning from infancy to puberty, weight remains a valid welfare indicator. During the initial welfare assessment, recording a weekly growth curve allows for tracking the normal development of the GA line compared WT animals. If a commercial line has been used as a background, there is also extensive literature available. Professional breeders provide growth curves for the strains they sell in datasheets, such as those for NZW rabbits, even with distinctions based on different breeding sites of the same breeder (58, 59), once more highlighting the importance of reporting breeding sites in the light of phenotype variability transparency, even when no formal substrain is to be considered under the genetic perspective. This availability of data allows for a double check: not only is the GA line characterized against WT littermates when available, but external control is also possible. Using growth curve data is recommended even during the maintenance phase of the GA line. This is because the small number of animals involved in the initial assessment may only provide partial information, and a larger sample size helps to establish a reliable key-performance indicator (KPI) that is species-specific and easy to monitor.

Identifying a minimum weight cut-off for choosing the appropriate weaning time can also be helpful at this stage. While there is variability based on genetic background and sex, weights above 500 grams at week 4 for both sexes, above 800 grams for females and above 900 grams for males and at week 5 are within standard for wild type individuals (58–61). Below this threshold, delaying weaning can help rabbits recover their size, increasing post-weaning survival expectations (62), as daily practice acknowledges already for laboratory rodents. Up to 8 weeks of age, daily gain weight – or its cumulative equivalent when larger weight checks intervals are considered – can be used to assess deviations between GA and wild type animals. An increment of 15–20 grams per day should be considered appropriate for the NZW, with different margins for other laboratory relevant strains, ranging from a minimum of 9–19 grams for the Polish and Dutch Belted (DB) and 24–25 grams for Californian and New Zealand Red (NZR) (63). In parallel with weight assessment, time to wean and progressive growth – and deviation from standards which are relevant for the welfare assessment of GA rabbits – can be identified and monitored using other biomarkers, like skeletal growth and, particularly, body length as already described in a previous study on New Zealand White rabbit and proposed for mice too (64, 65).

Once again, as rabbits transition to puberty and sexual maturity, weight remains a key biomarker. Although sexual maturity depends on the breed, body weight is generally more important than age, as is the case with other species (66–68). Small breeds can reach sexual maturity already at 4–5 months, medium breeds at 4–6 months, and large breeds at 5–8 months. Female NZW rabbits reach maturity around 5 months, while males mature at 6–7 months, with late individuals reported at around 8 or 9 months among females and males, respectively, (60, 61, 69). In this sense, any relevant deviation from growth rate curves and the presence of sexual milestones delays can be assessed against any genetic alteration, once again considering housing densities, proven to impact weight gain (70).

Beyond growth and compliance with developmental milestones, a few clinical conditions can be of relevance in this phase. On one hand, GI conditions arising due to the dietary re-arrangement in this phase should be checked for and distinguished from any GA related phenotype. For laboratory rabbits, this could be particularly relevant in cases where different diets are used for breeding and maintenance phases, or in cases where animals are bred at one Institute and sent for experiments to a satellite experimental facility or to another Institute. Also, transitioning into puberty and early adulthood can trigger some expected behavioral changes in young rabbits, including increased territoriality or aggressive behavior in both sexes (63). The recording of abnormal frequencies or severity of such behaviors should be carefully assessed to evaluate whether increased aggression might be intrinsically related to a genotype or due to an unfortunate selection of one or more founders. In the latter case, taking corrective actions by breeders’ selection, when possible, to reduce the incidence of unwanted behavioral traits can still rescue an aggressive phenotype that might other ways expand in the colony, impacting the overall severity of the model. On the other hand, specific clinical conditions related to the induced model may start to become clinically overt. Those must be assessed carefully with reference to the expected outcome of the genetic modification.

#### 2.2.2 Early adulthood, reproduction and breeding performances

Adulthood is the age when sexual maturity is fully attained, and for laboratory rabbits, based on different strains, young adulthood is the time when they are the most active and estrous cycle is regularized thus considered continuous in the doe (63). A core assessment of GA rabbits in their adulthood is represented by reproductive capabilities. Particularly, sterility, infertility and fertility rates, litter size, parental care and overall reproductive lifespan can all contribute to the welfare assessment of a GA strain.

The conditions of infertility in GA rabbits can be suspected when repeated attempts at mating do not result in pregnancy or birth of live kits. Standardized ultrasound pregnancy check at 14 days post-mating helps determine if there is sterility or intrauterine embryonal mortality. If reproductive problems are suspected or confirmed, it is essential to evaluate which sex, or genotype is contributing to the infertility. A standard approach to assessing this is to examine the effects of sex and genotype by swapping the breeders, for example, using a GA male with a WT female and vice versa. It is crucial to use proven fertile WT animals as controls to ensure the validity of the results. Consistent failure in reproduction of a specific sex and GA genotype in more than one subject is strongly suggestive of genetic modification impacting on fertility.

Pregnancy rates or more precisely kindling rates, critical measure of reproductive success, can be compared by assessing the performance of breeders with a specific sex and genotype. While average pregnancy rates for several rabbit strains and hybrids are available in the literature (71), on-site comparisons, such as comparing GA females to WT females used in the generation and maintenance of the same GA line, can provide more reliable data. This approach neutralizes potential confounding factors related to environmental and husbandry conditions that may influence reproductive performance (72).

When litter is given, litter size can serve as a quantitative parameter to evaluate any deviations between GA rabbits and WT counterparts. With a specific focus on laboratory rabbit strains, vendors should transparently report the average litter size of their commercial rabbits (61), which can then be used as a preliminary benchmark for comparison. In cases where litter size data is not publicly disclosed, a direct inquiry with the vendor is recommended. This inquiry should also address the type of mating performed, as the mating method, whether natural or by artificial insemination, can have a significant impact on breeding outcomes, including stillbirth rates and, consequently, litter size (73). Also, prolificacy, that is the litter size at birth (total and alive), can be assessed and should be recorded within a maximal interval of 24 h post-partum (74). This should always be balanced with genetic assessment of kits found dead as previously described. More granular assessment of breeding capabilities to catch significant deviations in GA strains can be achieved by calculation of the kindling rate (KR), that is the actual percentage of does with a given litter based on the number of inseminated does and thus more precise than the pregnancy rate, and subsequently of the kit index, product of the KR multiplied by the average LS (71). Those parameters could contribute to further understanding the presence of relevant deviations from WT animals but could be used even more effectively in the long-term maintenance of a GA colony, to track and identify any deviations from standard reproductive performances within the colony, contributing to corrective actions to rescue performances. Other specific animal-related parameters should be considered to interpret reproductive data against a possible GA-related phenotype, such as age of breeders with respect to expected breeding lifespan for both males and females (60, 61) and parity (55). All in all, it is important to consider that breeding performance and breeding lifespan can be tremendously affected by animal facility and husbandry specific factors including, among others, type of environment, diet and feed intake, seasonality and light cycles, temperature, hygiene status including microbiome in both sexes (35, 75–80). As such, divergence between GA animals and WT counterparts should be balanced against all the potential aforementioned co-factors, once more highlighting the importance of providing accurate, holistic metadata in the welfare assessment reports and when transferring one GA strain to a different institution.

To accurately balance the potential effect of genetic alteration versus spontaneous clinical conditions, a good understanding of spontaneous pathology of the rabbit during reproductive lifespan is crucial. As a matter of examples, ectopic gestation is described in NZW rabbit, with higher prevalence in does subject to artificial insemination, as well as uterine ischemia and fetal hypoxia and intrauterine growth restriction (81, 82). With regards to female reproductive tract spontaneous lesions, cysts are commonly recorded with high prevalence in the oviduct and ovary in NZW strain, while uterine cysts are the most identified in DB rabbits (83). Common background findings for the NZW male reproductive tract are, among others, tubular atrophy along with Leydig cells atrophy, squamous metaplasia in the prostate and seminal vesicles (84). A structured gynecologic clinical evaluation and instrumental pregnancy follow-up should be put in place to gather relevant, evidence-based information of the reproductive phases, allowing to establish normative values and understand potential GA related phenotypic deviations.

### 2.3 Aging rabbits and long-term assessment

Initial welfare assessment normally stops with animals reaching reproductive age, and later timepoints are recommended when late-onset phenotypes may be expected and should thus be carefully assessed consistently (1). Nevertheless, whenever possible and without the generation of dedicated animals, later timepoints should always be considered and analyzed. In other words, welfare assessment is a life-long pipeline with regards to the life of the colony, not of the individual subjects, and information should be collected from any individual of the colony as long as maintained in the facility, transitioning from early to middle and late adulthood. This approach allows for a constant, accurate supervision of the line, covering initial welfare assessment, refining of findings emerged in the initial welfare assessment, better understanding of the phenotype and timely identification of any deviations from original welfare assessment or unexpected late onset outcomes of the genetic manipulation. Aging effects can be assessed by considering breeders and retired breeders but also proper aging models, when the GA line has been created or is maintained for this purpose. In GA rabbit colonies, it is not infrequent to maintain effective breeders for extended periods, especially for males up to 5–6 years unless clinical conditions compromise welfare or productivity. This is an advantage, as it allows prolonged observation and long-term monitoring of possible late-onset phenotypes. Assessment of such animals allows us to consider several key traits of GA animals against their WT counterparts maintained as negative controls or using reliable literature data of the background strain as a complement, when available. Life expectancy for the rabbit is reported to be between 5 and 12 years, with shorter lifespan and variations based on different laboratory strains and husbandry conditions (60, 63, 85). When maintaining rabbits for several years, this parameter can be positively assessed to detect deviations regarding maximal average life expectancy. On the other hand, in laboratory models, when euthanasia may be scheduled at earlier timepoints, significant mortality below the minimum life expectancy should be considered as potentially suggestive of a GA related trait, when deviations from WT background baselines are evident. As already recommended for animals found dead, or euthanized due to severe welfare and health impairment, full necropsy and anatomopathological analysis is a crucial step to correctly identify underlying causes of such impairments, to reinforce or rule out the impact of the genetic alteration. To do so, knowing what to expect in terms of common findings, spontaneous lesions and clinical conditions of aging and geriatric rabbits is paramount, and broader veterinary practice can contribute to collect and disseminate knowledge, complementing data deriving from laboratory animal science. Clinical conditions and findings can include chronic renal failure, cardiovascular diseases, inflammatory or degenerative joint diseases, muscle wanting, cataract formation, orofacial masses and spontaneous lung lesions (85–87). In aging rabbits, neoplastic degeneration is well described in pet, production and laboratory rabbits and consistently from the second year of life (85, 86, 88–90). Common cancers include uterine adenocarcinoma, with metastatic potential to liver and lungs, thymoma, squamous epithelium tumors, lymphoma, mammary gland and skin tumors (91). Sufficient information is available to enable the description of an oncological profile as a function of age and sex and inheritance predisposition to specific types of cancer (e.g., uterine adenocarcinoma) in the rabbit (90), and its incidence and presentation in GA rabbits should be balanced against similar cases in WT counterparts. A comprehensive reference describing the spontaneous pathology observed in common laboratory rabbit strains, organized by organ system, is now available and provides important baseline information that may also support the comparative assessment of genetically altered rabbits (92). Criteria, key indicators and possible biomarkers to assess welfare impairment in aging rabbits are summarized in Table 4.

**Table 4:** Specific considerations for welfare assessment of aging rabbits.

High-level category	Criteria	What to Look For
Longevity and survival	Life expectancy and survival trends	Reduced lifespan, premature mortality, or deviations from expected strain and colony survival patterns compared with WT controls
	Late-onset phenotype emergence	Clinical signs or welfare impairments appearing during middle or late adulthood that were absent at younger ages
Clinical monitoring	General age-related clinical conditions	Evidence of chronic renal disease, cardiovascular disease, degenerative joint disease, cataracts, respiratory lesions, or other spontaneous aging-associated conditions
	Neoplastic disease surveillance	Development of tumors (e.g., uterine adenocarcinoma, thymoma, lymphoma, mammary, skin, or squamous epithelial tumors), including age- and sex-related incidence patterns
	Necropsy and histopathology findings	Pathological lesions identified at necropsy that may explain morbidity, mortality, or severe welfare impairment
	Any non-specific clinical conditions	To be assessed against expected effects of GA – aging model related phenotype and non-expected effect of GA – background related conditions versus GA-related outcome.
Reproductive aging	Long-term breeder performance	Declining fertility, reduced productivity, shortened breeding lifespan, or increased reproductive complications in aging breeders
Environmental and husbandry factors	Institution- and environment-related influences	Phenotypic or welfare changes potentially associated with microbiome, housing, diet, hygiene, or long-term facility-specific conditions
Colony-level long-term monitoring	Longitudinal colony database trends	Deviations over time in growth, productivity, lifespan, hematobiochemical values, or other welfare-related indicators compared with historical colony baselines* Requires GA line documentation and traceability and colony metadata.
	WT comparison and line drift assessment	Progressive divergence from WT controls or from original colony characterization suggestive of drift or environmental adaptation.

A refined approach to assessing the welfare of aging GA rabbits may consider implementing a frailty phenotype assessment. Frailty can be defined as a lack of resiliency in the face of external stressors (93), and more precisely in gerontology it refers to a state of increased vulnerability as consequence of cumulative decline in many physiological systems during a lifetime (94). The Fried frailty phenotype for humans proposes that frailty be defined as a clinical syndrome in which three or more of the five following criteria are present: unintentional weight loss, self-reported exhaustion, weakness (grip strength), slow walking speed, and low physical activity (95). Several approaches to assess frailty in animal models have been proposed for rodents, and for invertebrate models too (96–98). Companion dogs have also been assessed to develop a frailty phenotype that could provide tools to assess aging impact and related risks for both canine and human health, acknowledging the potential of pet dogs as translational models for human aging due to shared environments and diseases (99, 100). In the case of GA rabbits, developing strategies to assess frailty can contribute to a wider understanding of genetic modification impact and harmful phenotype, if any. Some relevant parameters identified for other species, also non rodents, and noninvasive to monitor, may be considered in the long-term assessment of aging WT rabbits and their GA counterparts. Multispecies, physically focused examples of frailty phenotype indicators associated with seniority and geriatrics, that is having reached 75% of life expectancy, could be: decreased or low physical activity levels, decreased or progressively poorer mobility, loss of muscle mass and weakness, decreased endurance, decrease in appetite and thirst, increase susceptibility to illnesses and age-related clinical conditions (98, 101–103). Proposed clinical and physiological indicators and possible biomarkers for a frailty phenotype of rabbits, adapted to species-specific traits of aging rabbits (85, 102), are summarized in Table 5. Given that frailty assessment remains largely unexplored in rabbits, model-oriented biomarkers for frailty phenotyping should be further pondered, developed and validated on a case-by-case basis.

**Table 5:** Proposed frailty phenotype for WT and GA rabbits.

High-level category	Criteria	What to look for
Frailty and Functional Decline	Physical activity and spontaneous behavior	Reduced overall activity, increased inactivity, reluctance to move, or decreased exploratory behavior
	Mobility and gait performance	Slower or unsteady gait, stiffness, impaired coordination, or difficulty with normal locomotion. Weakness of hindlegs; Loss of joint flexibility and poor self-grooming with evidence of neglected fur and/or impaired cecotrophy.
	Muscle mass and body condition	Progressive muscle wasting, loss of body condition, reduced strength. Body Condition Scoring consistent with very thin to cachectic states. Prominent iliac crests; sharply visible scapulae and vertebral processes.
	Endurance and fatigue resistance	Early exhaustion, reduced ability to sustain normal activity, or marked decrease in exercise tolerance.
	Appetite and hydration status	Decreased food and water intake and/or reduced feeding motivation, including decrease or loss of interest for multiple highly palatable food or food enrichment.
	Susceptibility to disease and stress	Increased frequency or severity of infections (e.g., pododermatitis, upper respiratory trait, mouth, GI tract), poor recovery after stressors, or heightened clinical spontaneous fragility.
	Sensory and functional decline	Reduced responsiveness, impaired grooming, or decreased engagement with environment, conspecifics or regular carers.
	Overall resilience to aging stressors	Reduced ability to maintain homeostasis under routine husbandry or environmental challenges: signs of distress including respiratory distress, eye bulging, vocalization in case of routinary handling, fur brushing, nail trimming, cage cleaning.
	Survival after frailty onset	Shortened time from first detectable functional declining signs to death or humane endpoint compared with WT counterparts; reduced post-frailty survival interval suggesting accelerated functional aging.

As a closing remark, the relevance of the original WT strain is, as extensively described, crucial for many aspects. However, it should be considered that GA lines maintained for long periods within a specific Institution will inevitably tend to deviate from the original background and may manifest characteristics (phenotypes, in the literal sense, not necessarily associated with any harm) specific to the facility of the Institution where they are housed secondary to institutional environmental parameters including microbiome among other factors (27, 77, 79). The very same GA line raised elsewhere could display different traits. This is why the so-called passports of GA lines are crucial, as they convey details about the environment and conditions in which welfare assessment and line characterization were performed. When maintained over the years at a specific institution, a GA colony becomes a tool for self-comparison. For physiological variables such as growth curves, productivity, life expectancy, hematobiochemical measures, or any other production-, welfare-, or research-relevant metrics, it is advisable to create a database at the institution. Over the long term, this allows deviations to be evaluated against the colony’s own historical data. This is particularly true for GA rabbits, considering that mitigating measures like genetic reset strategies such as regular backcross or cryo-recovery from repositories may be more difficult to implement than with rodents.

## 3 Practical, welfare, ethical and legal considerations

**Figure 1:**
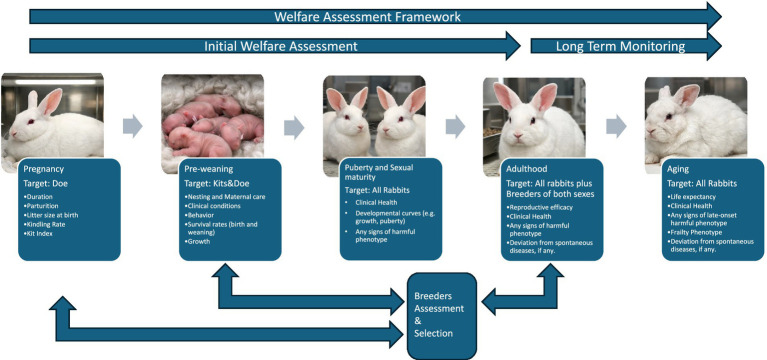
Schematic summary of the proposed welfare assessment framework for GA rabbits.

Correctly performing the initial welfare assessment and maintaining control of the local animal colony in the long term (Figure 1) are essential and require species-specific oriented approach, as experience of pain, suffering and distress is intrinsically linked to the specie’s behavior, neurodevelopment and physiology (104–106) as well as individual specific predisposition leading to the experience of varying levels of harm even in the same colony. It is essential to account for these nuances when designing welfare assessments (107).

**Figure 2:**
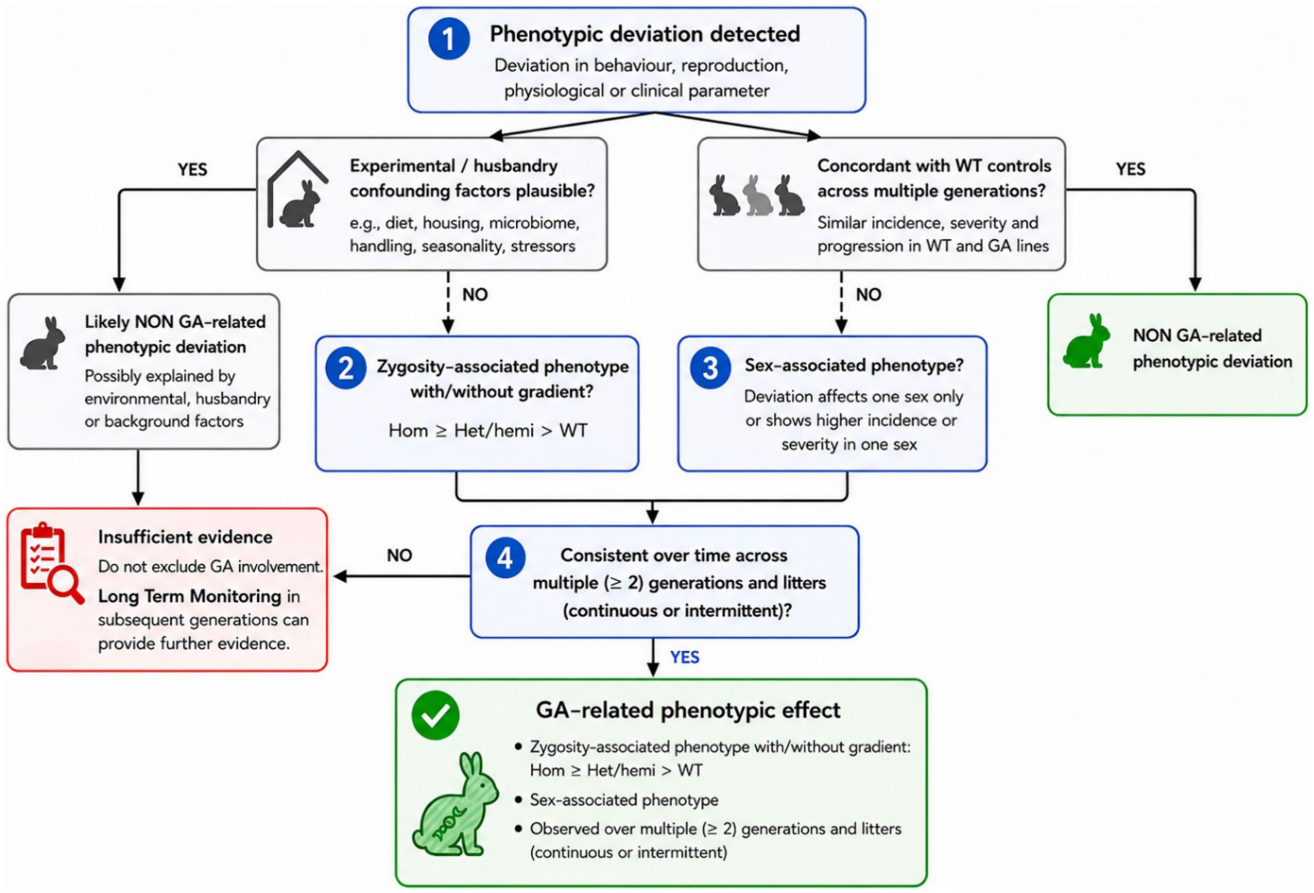
Decision tree for the welfare assessment of GA rabbits.

To determine the presence of a harmful phenotype, it is first necessary to identify the occurrence of relevant phenotypic deviations. As presented in the above paragraphs, different life stages and biological phases are associated with multiple indicators and biomarkers that can be used by involved personnel to monitor the animals for normality and detect, if any, relevant deviations, initially estimating the presence of a potential harmful trait and subsequently quantifying its actual severity. The detection of a deviation in behavioral, reproductive, physiological, or clinical parameters should prompt the question of whether the strain under assessment carries a GA-related phenotype. The answer is not always straightforward. Factors such as concordance with WT controls across multiple generations, or the plausibility of experimental and husbandry-related factors, support the likelihood of a non-GA-related condition. Conversely, a consistent genotype-associated pattern strongly supports the involvement of the GA construct. Examples include gradients of severity of phenotypes, with more severe signs in homozygous animals, equal or milder phenotypes in heterozygous or hemizygous animals, and absence of the phenotype in WT counterparts. A more complex interpretative scenario arises when a mild phenotype is also present in WT animals, but with increased severity in GA subjects. This may suggest either an amplifying effect of GA on spontaneous background condition or the unfortunate selection of a founder line intrinsically predisposed to a more severe phenotype. In such cases, comparison with additional founders, when available, may help clarify the origin of the deviation. Likewise, deviations affecting only one sex, or occurring with significantly higher incidence or severity in one sex, should raise suspicion of a GA-related effect. Persistent occurrence of the same deviation over time, even if intermittent, and reproducibility across multiple generations further support a causal association with the GA construct. Conversely, failure to reproduce a deviation across two or more generations reduces the strength of evidence for a GA-related effect, although it should not lead to complete exclusion of GA involvement. In these cases, long-term colony monitoring remains important, and subsequent generations should continue to be assessed for early recurrence of similar deviations. A simplified decision tree to orient toward or against GA-related deviations is represented in Figure 2.

Suspecting first and then confirming the presence of a harmful phenotype triggers legal requirements, ethical obligations, and the need for accurate measures to secure the welfare of the animals. This ensures compliance with regulatory frameworks (1) and highlights the importance of ongoing monitoring to address any emerging issues throughout the life of the colony. In the light of the 3Rs principle and its evolution and relevance for GA animals (108), refinement strategies like breeder selection, setting dedicated time points for biological milestones such as weaning or breeder’s set-up and retirement, and adjusting housing conditions, enrichment and species /strain specific preventive medicine programs are key to mitigating and minimizing suffering. Specific attention must be paid to humane endpoints, which should be determined considering both research goals and, especially for the maintenance of the colony, consistently with time points of phenotype onset, ensuring the animals’ needs are met throughout the whole process. Strategies to, e.g., cull animals before the onset of the harmful phenotype do not reduce the legal burden, as the risk of developing a late-onset harmful phenotype is enough to require project authorization (1), but it can reduce the burden in terms of welfare impairment, which should always be seen as the highest priority, whenever possible. At the intersection of refinement and reduction, understanding phenotypic deviations of the GA line from baseline values, as well as the behavior of the genetic mutation (e.g., dominance, recessivity, frequency), also helps predict productivity, a crucial factor for determining optimal colony size and reduce surplus animals. In terms of reduction, it’s crucial not to generate *ad hoc* animals solely for the purpose of assessing a line (1, 52). This highlights the importance of long-term assessment, evaluating every animal born and maintained in the breeding and experimental colony. Under EU regulation for example, at least 7 males and 7 females of the desired genotype are assessed against WT counterparts, while Switzerland requires gathering information from 100 individuals over 3 generations (if possible) for the entire lifetime and issuing a preliminary alert to authorities after recording 5 likely clinical cases (1, 52). The appropriate number of animals needed to identify clinical and welfare issues in a GA colony can vary depending on the type of mutation, its penetrance and frequency of expression in the genotype (1, 107) and the time at which the condition becomes overt, especially if late-onset phenotypes are expected and accounting for more individuals in the colony may thus be recommended as a precaution (1, 104). A further aspect of potential constraint in the light of Reduction when dealing with GA rabbits colonies resides in cryobiology limitations. Cryopreservation and revitalization of a GA line are valuable strategies to reduce unnecessary breeding and surplus animals during long lasting intervals between experimental projects (109, 110). Conversely to rodents for example, cryopreservation of rabbit embryos requires expensive equipment and producing large numbers of stored embryos requires the sacrifice of many donor females. Although alternative methods of embryo recovery (e.g., surgical or vaginal collection) are possible, such procedures produce fewer embryos. Not only in the light of overall reduction, but also to preserve genetic diversity, standardize strains and protect them from genetic drift and human error, frozen embryos are the main way for rabbit cryo-conservation (111). Sperm collection and cryopreservation can be used to obtain progeny after thawing and artificial insemination (AI) of females, nevertheless its reliability is still debatable, and results after AI with rabbit frozen semen are still inconstant (111, 112). Interestingly, tests have been carried out in the rabbit to evaluate the effects of the cryopreservation program on the genome and phenotype of the rederived rabbits, showing transgenerational effect, affecting lipid metabolism and growth, but being apparently innocuous on reproductive efficiency (113). The impact of such consequences should be accurately considered if after revitalization the GA line may show unexpected deviations from the original cryopreserved strain.

Finally, the ethic debate on inducing genetic alterations in animals and objection to such manipulations go beyond welfare *per se*, including the arguments that modifying animals’ genome equals to playing God, is unnatural, violates their integrity and entelechy turning them into instruments (114, 115). Notably, as highlighted by Bovenkerk, such arguments “beyond welfare” appear to relate to broader conceptions of the “good life,” linked to the intrinsic value we recognize to animals and the subjective idea that we have of animals and humans, and carries values within (114). While it is by no means the scope of the authors to deny the scientific value of *in vivo* research, nor to question the scientific importance of transgenic animal models, the responsible generation and use of GA animals implies acknowledging the risk of major welfare impairments and accepting the responsibility, shared by researchers, veterinarians, animal welfare officers and competent authorities, within the perimeter of their specific competence, to not only minimize the negative experiences but to proactively work to secure and promote positive welfare by going beyond minimal legal requirements and building a life worth living for experimental animals. The whole pipeline of initial and long-term welfare assessment presented here and summarized in Figure 1, should be seen as a shared strategy to contribute to a *life worth living* for GA rabbits, going beyond the mere principles of the 3Rs and its check-box mentality (116) and the non-maleficence framing of the five freedoms (117).

## 4 Limitations and future validation needs

The proposed framework is primarily based on literature synthesis and expert opinion. Due to the limited availability of GA rabbit-specific data, several proposed criteria and assessment approaches were extrapolated from related evidence sources, including laboratory rabbit medicine, production rabbit welfare literature, pet rabbit clinical assessment, toxicology control protocols and, from a methodological perspective, established welfare assessment approaches applied to other GA species. Consequently, the proposed indicators provide a practical and biologically informed basis for welfare assessment in GA rabbits, while remaining open to further refinement as additional evidence becomes available. In addition, thresholds and interpretation of indicators may vary substantially according to rabbit strain, sex, genotype, age and facility-specific environmental factors, including microbiome status, housing conditions, diet and husbandry practices. This framework is therefore intended as a flexible and adaptive guide rather than a prescriptive scoring system uniformly applicable across all settings. In this context, the framework provides a structured basis for pilot implementation and prospective validation in GA rabbit colonies, to assess feasibility, inter-observer reliability, sensitivity to welfare impairment, and applicability across different regulatory settings, genetic backgrounds, GA disease models and husbandry systems.

## 5 Conclusion

Understanding the effects of genetic manipulation on a living being is a task requiring a complex, accurate and meticulous assessment, covering initial developmental milestones, where overt effects become often evident, together with a long-term monitoring to better understand the whole effects of the alteration on a wider number of animals and in later life stages. Often, the effects of transgenesis can be null or under threshold, other times they can negatively impact the wellbeing and health of GA animals inducing different levels of harm and severity, triggering ethical and legal consequences that must be appropriately and responsibly managed. With regards to GA rabbits, knowing the peculiarity of the species is a key factor to identify the most appropriate indicators and biomarkers to detect any deviations from baseline throughout all life stages of the rabbit, thus to identify preventive and mitigation measures to reduce the burden, and to develop species- and model-targeted strategies to improve the quality of life of GA rabbits, while securing rigorous scientific research, experimental data, legal and ethical compliance. This harm-action- benefit strategy acknowledges the GA animals’ dignity.
